# Early Public Response to Influenza A(H7N9) Virus, Guangzhou, China, May 30–June 7, 2013

**DOI:** 10.3201/eid2007.131155

**Published:** 2014-07

**Authors:** Jun Yuan, Qiuyan Liao, Eric H.Y. Lau, Zhi Cong Yang, Xiao Wei Ma, Jian Dong Chen, Yan Hui Liu, Chang Wang, Xiao Ping Tang, Yu Fei Liu, Gabriel M. Leung, Xin Cai Xiao, Richard Fielding, Ming Wang, Benjamin J. Cowling

**Affiliations:** Guangzhou Center for Disease Control and Prevention, Guangzhou, China (J. Yuan, Z.C. Yang, X.W. Ma, J.D. Chen, Y.H. Liu, C. Wang, Y.F. Liu, X.C. Xiao, M. Wang);; The University of Hong Kong, Hong Kong, China (Q. Liao, E.H.Y. Lau, G.M. Leung, R. Fielding, B.J. Cowling);; The Eighth People’s Hospital of Guangzhou, Guangzhou (X.P. Tang)

**Keywords:** public, influenza, influenza virus, influenza A(H7N9) virus, subtype H7N9, live poultry markets, China, Guangzhou, behaviors, attitudes, urban, semirural, viruses, chickens

**To the Editor:** On May 15, 2013, surveillance in live poultry markets (LPMs) identified an influenza A(H7N9) virus–infected chicken in Guangzhou, the capital of Guangdong Province, China. During May 30–June 7, 2013, we conducted a population-based survey in Guangzhou to investigate changes in the public’s buying behavior at LPMs and to determine the public’s attitude toward potential implementation of specific interventions against avian influenza in LPMs. Behaviors and attitudes in 3 residential areas of Guangzhou were compared: urban districts, Conghua (a semirural area), and Zengcheng (a semirural area where subtype H7N9 infection had been detected in an LPM on May 15, 2013). These locations were chosen to compare possible urban–rural differences in live poultry exposure, as observed in an earlier study (*1*), and to assess the effect of epidemic proximity on the public’s attitude and behavior.

Study participants were recruited by using the Mitofsky–Waksberg 2-stage sampling method (*2*). First 120, 60, and 60 telephone prefixes in urban districts, Conghua, and Zengcheng, respectively, were randomly selected. Then for each prefix, telephone numbers were randomly generated and called until 5 households were successfully recruited. Within each household, the person whose birthday was closest to the interview date and who was ≥15 years of age was invited to participate in the telephone interview. Using a standardized questionnaire, we collected demographic information and information on behavior related to buying live poultry from LPMs, attitudes toward measures for reducing avian influenza transmission in LPMs, and perceived risk for infection from LPMs.

Of 1,930 persons recruited, 1,196 (62.0%) completed the interview. Information on age was missing for 19 persons, so they were excluded; thus, a total of 1,177 persons were included in the analysis. Responses from the 3 residential areas were generally comparable, with the exception that respondents from urban districts reported higher levels of education and personal income (Technical Appendix). Compared with the overall Guangzhou population (*3*), the respondents were slightly better educated and less likely to be single (Technical Appendix).

We used logistic regression models (*4*) adjusted for age, sex, and education level to calculate the percentages and 95% CIs related to buying live poultry from LPMs, attitudes, and risk perception in each area and for the sample as a whole. During the 2 months before the survey, ≈33.5% (95% CI 29.7%–37.5%) of the sampled households bought live poultry from LPMs at least once a week (Table). The number of households that bought live poultry on a weekly basis was substantially lower in Zengcheng than in urban areas. After the epidemic in Zengcheng was announced, 59.1% (95% CI 55.1%–63.0%) of all respondents reported buying less poultry or having completely stopped buying live poultry. Compared with respondents in the other 2 areas, Zengcheng respondents were more likely to report a reduction in buying (Table).

**Table:** Public attitudes and behaviors in response to influenza A(H7N9) virus, Guangzhou, China, May 30–June 7, 2013*

Characteristic	% (95% CI) persons with characteristic			
	Urban districts, n = 594	Semirural districts		Total, N = 1,177
		Conghua, n = 283	Zengcheng, n = 300	
Household purchase of live birds from LPMs				
Weekly to monthly	21.2 (17.6–25.2)	29.7 (23.8–36.5)	19.7 (14.7–25.8)	21.6 (18.5–25.0)
At least weekly	34.1 (29.7–38.7)	29.5 (23.2–36.6)	**23.3 (18.1–29.6)**	33.5 (29.7–37.5)
Buying less/stopped buying because of A(H7N9) epidemic	58.7 (54.0–63.3)	56.3 (49.3–63.2)	**71.6 (64.8–77.5)**	59.1 (55.1–63.0)
Attitude (agree/strongly agree) toward control measures				
Introducing 1 or 2 market rest days per month	88.7 (85.4–91.4)	90.5 (85.9–93.7)	91.2 (87.1–94.0)	89.7 (87.6–91.5)
Closing LPMs	20.8 (17.3–24.7)	16.2 (12.0–21.4)	**31.1 (25.4–37.3)**	21.1 (18.1–24.4)
Perception (agree/strongly agree) of risk from LPMs				
Live animals sold in markets are a risk to human health	34.0 (29.8–38.5)	33.3 (26.9–40.4)	38.2 (32.0–44.7)	34.3 (30.7–38.1)
Poor market hygiene is main cause of avian influenza transmission	68.5(64.0–72.7)	73.1 (66.9–78.6)	68.3 (61.7–74.4)	69.0 (65.2–72.5)
Likelihood (likely/very likely) of getting sick as a result of buying live birds from LPMs	18.7 (15.5–22.4)	**9.9 (6.8–14.1)**	**30.0 (23.8–37.0)**	19.1 (16.3–22.3)

*All values are weighted by population age and sex and adjusted by sample age, sex, and education level, using logistic regression models. Boldface indicate that attitudes/perceptions in Conghua or Zengcheng were significantly different (p<0.05) from those in urban districts. LPMs, live poultry markets.

Most respondents expressed support for the policy of introducing 1 or 2 monthly market rest days in Guangzhou, but only 21.1% (95% CI 18.1%–24.4%) agreed with complete closure of LPMs (Table). Zengcheng respondents were more likely to agree on closure of LPMs. Approximately one third of the respondents agreed that live animals sold in LPMs posed risks to human health, and more than two thirds agreed that avian influenza transmission was due to poor market hygiene. However, only 19.1% (95% CI 16.3%–22.3%) of the respondents perceived that they would be likely/very likely to get sick from buying live poultry from LPMs. Perceived risk from buying was highest in Zengcheng and lowest in Conghua (Table).

Although there were no cases of human A(H7N9) infection in Guangzhou at the time of study, >50% of respondents reported a reduction in buying live poultry from LPMs after the A(H7N9) epidemic in China was officially announced (*5*). Information on A(H7N9) virus may have motivated a population-level change in live poultry buying habits; however, such change is likely to be temporal (*6*). Detection of A(H7N9) virus infection in poultry may have further raised risk awareness and prompted behavioral change in the local area (*7*). However, respondents seemed to prefer measures such as monthly market rest days in LPMs rather than the more extreme measure of complete LPM closure. This finding suggests that the progressive introduction of measures to control avian influenza risk in LPMs would be more acceptable to the public. Although the risk from sales of live animals in LPMs was generally ignored, poor market hygiene was commonly accepted as a cause of avian influenza virus transmission. The public should be educated about the risk from sales of live animals in markets.

This study was limited by its reliance on self-reported data, making results potentially subject to social desirability bias. In addition, stratified rather than simple random sampling was used to recruit participants, so the sample may not be representative of the general population of Guangzhou. Furthermore, the study was conducted before any human cases of A(H7N9) infection were confirmed in Guangzhou, so the results may not apply to other parts of China with confirmed human cases. The results should, however, guide public health responses in regions neighboring epidemic areas.

Technical Appendix Characteristics of respondents to a survey of attitudes and behaviors in response to influenza A(H7N9) virus, Guangzhou, China.
