# Supplementary material for: Early Public Response to Influenza A(H7N9) Virus, Guangzhou, China, May 30–June 7, 2013
Source: Emerg Infect Dis. 2014 Jul;20(7):1238–40. doi: 10.3201/eid2007.131155 (PMC4073867; doi:10.3201/eid2007.131155)
Supplement: Technical Appendix — Characteristics of respondents to a survey of attitudes and behaviors in response to influenza A(H7N9) virus, Guangzhou, China. [file 13-1155-Techapp-s1.pdf]

Article DOI: http://dx.doi.org/10.3201/eid2007.131155

Early Public Response to Influenza A(H7N9) Virus, Guangzhou, China, May 30–June 7, 2013

Technical Appendix

Technical Appendix Table. Characteristics of the population of Guangzhou, China, and of survey respondents in urban districts and semirural areas of Guangzhou, May 30–June 7, 2013

Characteristic	% (95% CI) participants by area: Urban districts, n = 594	Conghua, n = 283	Zengcheng, n = 300	Total, N = 1,177	% Guangzhou population*	Difference between residential areas (p)†	Effect size‡
Sex							
F	59.1 (55.0–63.1)	53.7 (47.7–59.6)	57.0 (51.2–62.7)	57.3 (54.4–60.1)	48.0	0.320	0.19
M	40.9 (36.9–45.0)	46.3 (40.4–52.3)	43.0 (37.3–48.8)	42.7 (39.9–45.6)	52.0		
Age group, y							
15–34	35.5 (31.7–39.5)	34.3 (28.8–40.1)	41.7 (36.0–47.5)	36.8 (34.0–39.6)	49.1	0.154	0.25
35–54	40.6 (36.6–44.6)	43.5 (37.6–49.5)	40.7 (35.1–46.5)	41.3 (38.5–44.2)	34.8		
≥55	23.9 (20.5–27.5)	22.3 (17.6–27.6)	17.7 (13.5–22.5)	21.9 (19.6–24.4)	16.1		
Education							
Primary or below	12.3 (9.8–15.2)	19.8 (15.3–24.9)	18.0 (13.8–22.8)	15.6 (13.5–17.7)	17.7	<0.001	0.34
Secondary	46.1 (42.1–50.2)	51.9 (46.0–57.9)	53.3 (47.5–59.1)	49.4 (46.5–52.3)	61.7		
Tertiary or above	41.1 (37.1–45.2)	27.2 (22.1–32.8)	27.7 (22.7–33.1)	34.3 (31.6–37.1)	20.6		
Unknown	0.5	1.1	1.0	0.8	0		
Marital status							
Single	14.6 (11.9–17.7)	13.8 (10.0–18.4)	18.0 (13.8–22.8)	15.3 (13.3–17.5)	32.4	0.316	0.36
Married or formerly married	85.2 (82.1–87.9)	85.5 (80.9–89.4)	82.0 (77.2–86.2)	84.5 (82.3–86.5)	67.6		
Unknown	0.2	0.7	0.0	0.3	0		
Monthly income (US$)§							
≤163	18.9 (15.8–22.2)	29.3 (24.1–35.0)	23.7 (19.0–28.9)	22.6 (20.2–25.1)	Not known	<0.001	
163–488	26.6 (23.1–30.3)	35.0 (29.4–40.9)	32.7 (27.4–38.3)	30.2 (27.5–32.9)	Not known		
488–813	20.9 (17.7–24.4)	16.3 (12.2–21.1)	12.0 (8.6–16.2)	17.5 (15.4–19.8)	Not known		
>813	20.2 (17.0–23.7)	5.3 (3.0–8.6)	9.0 (6.0–12.8)	13.8 (11.8–15.9)	Not known		
Unknown	13.5 (10.8–16.5)	14.1 (10.3–18.7)	22.7 (18.1–27.8)	16.0 (13.9–18.2)	Not known		

*Population structure was obtained from the 2010 Guangzhou census data (http://www.gzstats.gov.cn/tjgb/glpcgb/201105/t20110517_25227.htm [in Chinese]).
†p values were based on χ^2 test.
‡Cohen's effect sizes w were calculated using the formula $w=\sqrt{\sum_{i=1}^{m}(p_0(i)-p_1(i))^2/p_0(i)}$, where $p_0(i)$ and $p_1(i)$ represent the observed proportions in the i'th category from the population (based on the 2010 Guangzhou census data) and the total sample, respectively.
§≤US$163, US$163–488, US$488–813 and >US$813 are approximate to categories of ≤1,000 Chinese Yuan (CNY), 1,000–3,000CNY, 3,000–5,000CNY, >5,000CNY (1US$ = 6.15CNY).
